# The Data Tags Suite (DATS) model for discovering data access and use requirements

**DOI:** 10.1093/gigascience/giz165

**Published:** 2020-02-07

**Authors:** George Alter, Alejandra Gonzalez-Beltran, Lucila Ohno-Machado, Philippe Rocca-Serra

**Affiliations:** 1 University of Michigan, ICPSR 330 Packard Street, Ann Arbor MI 48104, USA; 2 Science and Technology Facilities Council, Scientific Computing Department, Rutherford Appleton Laboratory, Harwell Campus, Didcot, 0X11 0QX, United Kingdom; 3 University of California, San Diego, Division of Biomedical Informatics, 9500 Gilman Dr. MC 0728, La Jolla CA 92093-0728, USA; 4 Oxford e-Research Centre University of Oxford 7 Keble Road, Oxford, OX1 3QG United Kingdom

**Keywords:** data access, data use, data discovery, metadata, confidential data

## Abstract

**Background:**

Data reuse is often controlled to protect the privacy of subjects and patients. Data discovery tools need ways to inform researchers about restrictions on data access and re-use.

**Results:**

We present elements in the Data Tags Suite (DATS) metadata schema describing data access, data use conditions, and consent information. DATS metadata are explained in terms of the administrative, legal, and technical systems used to protect confidential data.

**Conclusions:**

The access and use metadata items in DATS are designed from the perspective of a researcher who wants to find and re-use existing data. We call for standard ways of describing informed consent and data use agreements that will enable automated systems for managing research data.

## Background

The vast amounts of data generated by researchers in many scientific disciplines hold potential discoveries extending beyond the work of those who created them. This is only possible if data can be discovered, accessed, and made available for reuse. The bioCADDIE Project [1], which was funded by the NIH Big Data to Knowledge Program (BD2K) [2] to create a way for researchers to search across all types of biomedical data, recognized that access conditions are an important part of data discovery. Researchers, when asked how they would use a data discovery index, emphasized their need for information about the conditions and methods for retrieving datasets of interest.

The access conditions imposed on researchers who reuse existing data are part of a chain of agreements intended to protect research subjects. An increasing number of research studies require data that may present risks to privacy if they are not protected. Human subjects may be exposed to re-identification from their genomes [3], geographic locations [4], or clinical data [5], and pieces of information that may be innocuous in isolation can allow re-identification when combined, particularly when linked to other datasets. A wide range of procedures and technologies are being deployed to allow researchers to analyze these data while protecting the rights of research subjects [6–9]. Researchers are aware that protecting confidential information imposes costs on them, and they want to know what to expect when it comes to data access and reuse conditions.

Researchers can only find the access conditions governing a dataset if those conditions are included in metadata describing the data. The bioCADDIE Project designed a new metadata schema called the Data Tags Suite (DATS) [10, 11] for its prototype data discovery index, DataMed [12]. Because the bioCADDIE team was tasked with indexing all kinds of NIH data, DATS needed to apply to a wide range of scientific domains. The bioCADDIE Descriptive Metadata Working Group reviewed existing metadata schemas and analyzed use cases collected from researchers. DATS was created with core elements that cover the basic information in any dataset and extended elements for specialized data types. DataMed [13] was designed with tools for mapping other metadata schemas into DATS. As this is being written, DataMed indexes 15 data types with >2 million datasets from 75 repositories.

The DATS standard continues to influence the development of metadata and data discovery tools. DATS is the core metadata specification of the Biomedical Research Computing System (BRICS) [14], which is used in a number of NIH data repositories [15]. DATS has also become a reference standard used in the development of other metadata schemas such as Bioschemas [16] and the W3C Data Catalog Vocabulary (DCAT) [17]. It is currently being evaluated to provide the underlying model for the EU Innovative Medicine Initiative FAIRplus data catalogue [18] to index fundamental, translational, and clinical trial data.

This article describes elements in DATS providing information about data access, data use conditions, and informed consent. The “access and use” metadata items in DATS are designed from the perspective of a researcher who wants to find and reuse existing data. We focus on the authorization to use a dataset, and we do not attempt to describe the rules used to classify data as confidential or the characteristics of data that make them sensitive. We also do not examine technical aspects of authentication, i.e., confirming the identity of the researcher. Because DATS was created for use in a data discovery index, we emphasize the impact of data protection procedures on data users. However, these procedures are part of a larger environment around patient privacy protection, and this article puts DATS metadata into the context of the administrative, legal, and technical systems used to protect confidential data.

## Protecting Confidential Research Data

Data providers try to balance the benefits of facilitating access to existing research data with their obligation to protect information provided by research subjects [19]. Datasets vary in the level of risk that subjects can be re-identified and in the amount of harm that subjects would suffer if their confidential information became known. Data providers have a range of data protection measures that differ both in their effectiveness and in the costs that they impose on researchers. The most burdensome and costly types of data protection are normally for data that pose the greatest risks to research subjects [7, 20, 21].

Ritchie [22] proposed a framework for protecting confidential data that is known as the “Five Safes” [23].

- Safe data: Modify the data to reduce the risk of re-identification of subjects.
- Safe projects: Review and approve designs of proposed research projects.
- Safe settings: Isolate the data in a secure physical location or by applying secure remote access technologies.
- Safe people: Require legal agreements that commit researchers to protecting confidential information. Train researchers in best practices.
- Safe outputs: Review analyses and other products before releasing them to researchers.

These headings describe a toolkit from which data administrators select a combination of measures appropriate for the disclosure risks in a particular dataset [24]. For example, a national sample of health interviews may be released after data masking procedures (“safe data”), such as “top coding” income into an open-ended category to make very wealthy individuals less identifiable. In contrast, health histories of patients with a specific disease in a limited geographic area are much more difficult to de-identify. Patient records may be released for only approved types of research (“safe projects”) through a secure remote access system (“safe settings”) under a formal data use agreement (“safe people”). Additionally, some health care institutions only permit release of analyses performed on their data after review (“safe outputs”). The challenge for a data discovery index is capturing those aspects of the data protection that impose costs on prospective data users, and potentially displaying only datasets that users may be authorized to use. The user should be able to filter results according to his/her ability to conform to the authorization criteria.

## DATS Access Metadata

The bioCADDIE Project invited an international group of advisors to participate in an Accessibility Metadata for Datasets Working Group to recommend metadata describing how researchers gain access to data. This group identified 3 processes in accessing data for reuse: authorization, authentication, and access method.

### Authorization

Obtaining permission from the party that owns or is responsible for protecting the data is an important step. A range of checks can be done for credentialing a user, which may take milliseconds or stretch into weeks (see Table 1). Conditions for authorization are spelled out in “data use agreements” (DUAs) that are based on study consent forms, HIPAA authorization forms, and other documents. Some data created for public use may not require any kind of permission (open access), but confidential data are protected by formal authorization agreements, which are called “licenses” in DATS (see below). For example, the U.S. Health Insurance Portability and Accountability Act (HIPAA) defines 2 standards for disclosing protected health information: “de-identification” and development of “limited data sets” [25]. Limited data sets are only available under a DUA because they contain information that increases the risk of re-identifying individuals (several authors have shown that individuals can be re-identified in “de-identified” data; see, e.g., [5]). The most restrictive authorization procedures are designed to limit data access to “safe people” (controlled access) who will respect the rights of research subjects and patients. Higher levels of security also come with a price. Obtaining institutional signatures on legal agreements is burdensome and reduces reuse of data [20, 26]. For electronic health record data, researchers are typically not the signatories of DUAs: this is usually reserved for institutions. Dyke et al. [27] propose registration with self-declaration of qualifications, purpose, and commitments as a level of protection between open access and authorization under formal agreements. The Working Group identified 6 common types of authorization.

**Table 1:** Descriptors for Authorization

Authorization type	Description
None	Not covered by a DUA
“Click through” online license	Users must agree to an online agreement without providing additional identification
Registration	Users must register before access is allowed and agree to conditions of use. Registration information may be verified
DUA signed by an individual	An agreement signed by the investigator is required. DUAs may require additional information, such as a research plan and an IRB review (see discussion of licenses below)
DUA signed by an institution	An agreement signed by the investigator's institution is required. DUAs require additional information, such as a research plan and an IRB review (see discussion of licenses below)

DUA: data use agreement; IRB: institutional review board.

Authorization procedures have implications for the accountability of the data user and timelines for accessing the data. For example, if researchers want to remain anonymous, they can only access datasets labeled with Authorization Types “None” or “Click through.” If they are not affiliated with an institution, they are not eligible for DUAs covering many types of biomedical data. Conditions in the DUA impose additional hurdles before the researcher will be able to use the data. Approval by an institutional review board (IRB) is often required, and the researcher may need to show that the purpose of the research is consistent with the consent forms signed by subjects in the study. Authorization is thus complicated, and a researcher may be allowed to use a particular dataset for one purpose (e.g., cancer research) but not another (e.g., a study on ancestry) (see the discussion of licenses below).

A data repository may use one or more of these authorization types. The Inter-university Consortium for Political and Social Research (ICPSR) [28], which is the oldest repository of social science data in the United States, has examples of all 5 authorization types among the studies in its collection.

### Authentication

When data are accessed online, many data repositories require some kind of login process to identify the user (see Table 2). Even when the data are not covered by a license, the user may need to create a username and password for access (i.e., registered access). Access to confidential data may require multi-factor authentication controls involving a second type of identification, such as a telephone number or dedicated IP address. Researchers who plan to automate harvesting of data from multiple sources are especially interested in authentication procedures. Three types of authentication were listed by the Accessibility Metadata for Datasets Working Group.

**Table 2:** Descriptors for Authorization

Authentication type	Description
None	No authentication required
Simple login	Single-factor login or the use of an authentication key or registered IP address is required
Multi-factor login	Multiple-factor login using a combination of IP address, password protection, authentication key, or other forms of authentication

### Access Method

Data repositories may protect confidential data by only allowing access in a physical or virtual “safe setting” (see Table 3). Researchers who want to use highly sensitive data may need to travel to a secure “enclave,” such as the Census Bureau's Research Data Centers [29] and the Veterans Health Administration VINCI system [30], or submit program code to be executed by the data repository (“remote service”) [6]. An increasing number of data providers allow researchers “remote access” to computers in a secure data center, and this is the model selected by the NIH-funded AllofUs Research Program [31]. Researchers working in these “virtual data enclaves” see a standard operating system, as they would on their local computer, but they cannot download data to their local machine [32, 33]. They are using a virtual machine launched from their local computer but actually operating on the remote system. An example of this is Vivli [34], a new platform for sharing clinical research data, which connects a data repository to a secure cloud-based workspace. When researchers are required to perform all of their analyses on a computer controlled by the data provider, the provider also has the option of examining and approving results before sending them to the researcher (“safe outputs”).

**Table 3:** Descriptors for Access

Access method	Description
Download	The data are available for download. A license may be required
API	Interaction with the data may be automated via defined communication protocols, i.e., APIs
Remote access	Users may access the data in a secure remote environment (“virtual data enclave”). Individual-level data may not be downloaded, only approved results
Remote service	A user may submit program code or the script for a software package to be executed in a secure data center. The remote site returns outputs. It may perform a review before releasing the results
Enclave	Access is provided to approved users within a secure facility without remote access. Results may remain at the enclave or be released after review

## DATS Use Metadata

A variety of terms are used to refer to legal agreements between data providers and users, including “data use agreement” (DUA), “data access agreement,” “material transfer agreement,” and “non-disclosure agreement.” In the “open data” world these agreements are called “licenses.” For example, Creative Commons licenses are used by openICPSR [35], figshare [36], and other data repositories for open access to data [37]. However, “license” usually implies access to the commercial value of an invention or software, and we prefer the term DUA for the agreements between data providers and users, especially for confidential data.

**Figure 1:**
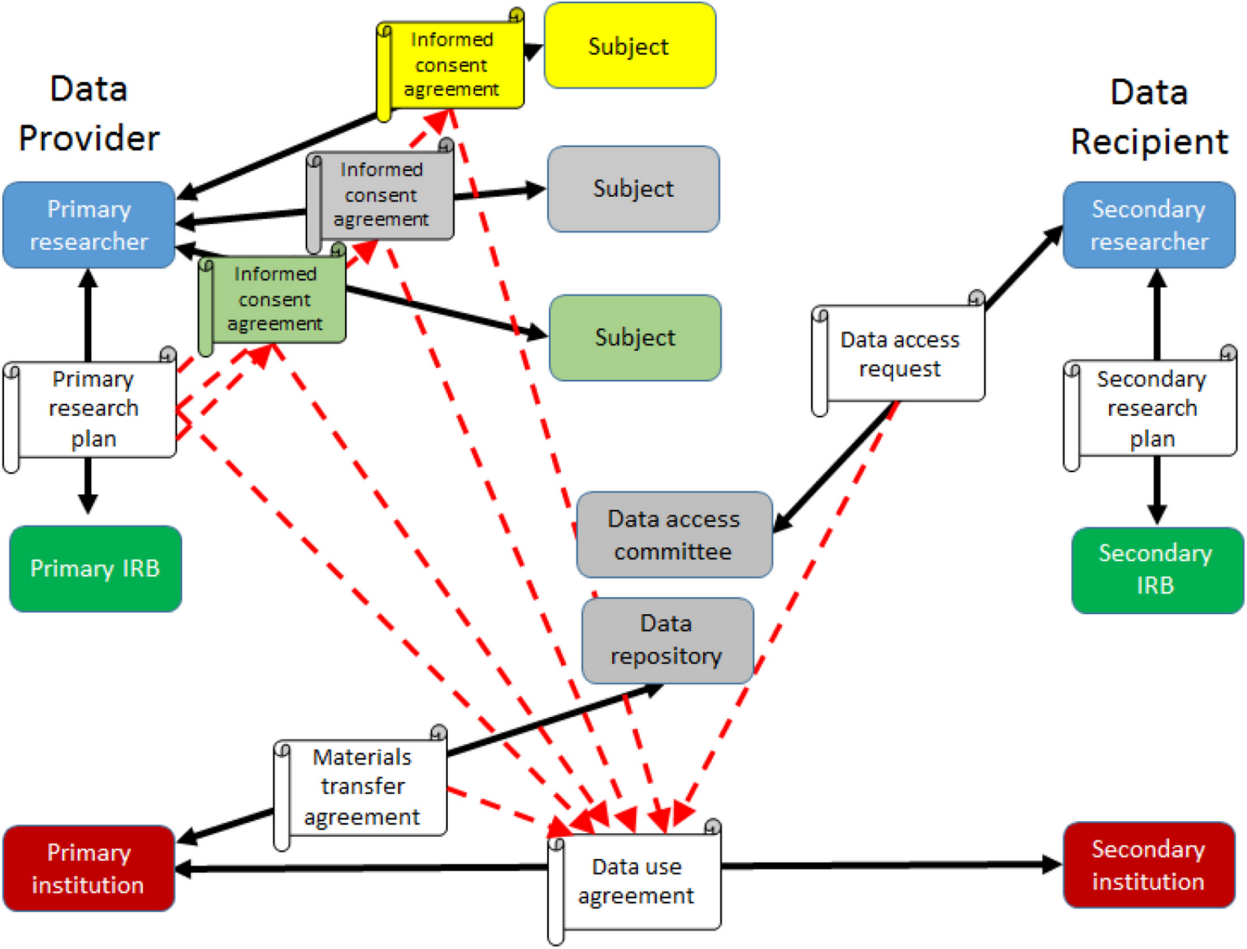
The network of agreements from data collection to data sharing. Solid lines connect parties in legal documents; dashed lines show agreements that are implicated in later documents. Documents are shown in white. Colors show roles and organizations.

DUAs are often lengthy agreements that inherit conditions from a number of earlier documents involving several different parties (see Fig. 1). DUAs include provisions describing allowed uses, limitations, and requirements: what analyses may be conducted, how long the data may be used, and the ways in which it must be returned, destroyed, or discarded after use. Solid lines in Fig. 1 connect parties in legal documents. Dashed lines show agreements that are implicated in later documents. DUAs typically require data users to obtain IRB approval from their home institutions, which may impose additional conditions on the data user's research plan. The bioCADDIE project did not attempt to create a comprehensive ontology of conditions found in DUAs, but DATS has been designed to take advantage of current efforts to use relevant ontologies when available. To understand the metadata needed to describe an agreement for reusing data, we outline how these agreements are created and administered. We focus here on the most complex case: agreements for data with some risk of harm to research subjects or patients.

Most academic research involving human subjects requires prior approval of a research plan by an ethical review committee or an IRB. In the United States, federal regulations mandate IRB approval for all research sponsored by NIH, NSF, and some other agencies [38], and most universities and other organizations require IRB review for all research involving human subjects. IRBs are responsible for protecting human subjects from the risks posed by research, which they do by approving and monitoring compliance with research plans. The research plan will include a description of documents and procedures for obtaining informed consent from research subjects involved in the study. The terms of the informed consent apply to all future research with these data, and an IRB should also review plans for sharing data resulting from the study. Researchers who analyze confidential data from a data repository are also expected to obtain approval from an IRB at their institution. However, IRBs do not make agreements with researchers at other institutions to share confidential data.

Transactions involving confidential research data are conducted by officials who are authorized to make agreements for the institution, such as a research administration officer. Most universities assert that research data belong to the institution, not to the researcher. If the research was sponsored by a funding agency, the “grantee” is the university, and universities see ownership of data resulting from external funding as part of their obligation to assure compliance with the terms of the grant or contract. Because confidential data also pose a risk to the reputation of the institution and possibly legal liability, universities are especially motivated to monitor the agreements surrounding them. This also applies to the institutions of researchers who request confidential data, and many universities will not allow faculty or staff to sign DUAs. Because data providers want the recipient university to be responsible for the management of confidential data, DUAs are typically signed by university officials on both sides.

DUAs often include a variety of conditions that were not explicitly included in the informed consent agreement. For example, some agreements include detailed requirements about data storage and computer systems. Agreements may require data to be stored offline and isolated from the Internet or be encrypted. Data recipients are often required to inform the data provider about any publications resulting from their secondary research. In some cases, the data provider insists on reviewing articles before they are submitted for publication or public presentation. Most data providers require a research plan describing how the data will be used, which may be reviewed by a panel of experts. For instance, researchers who ask for data from NIH's Database of Genotypes and Phenotypes (dbGaP) repository must submit a Data Access Request for review by a Data Access Committee [39, 40]. The Data Access Request becomes part of the DUA and limits the recipient to the approved analyses.

“Dynamic consent” is a rapidly developing practice with important implications for data access [41]. Until recently, informed consent agreements were static documents signed by a research subject when data were collected. There is a strong movement to give research subjects ongoing control over the use of their data [42]. Subjects may be able to withdraw consent at any time, and several new technologies allow them to choose which research projects can use their data [43, 44]. Dynamic consent conforms to the spirit of the European Union's General Data Protection Regulation (GDPR), but the GDPR exempts scientific research from rules giving subjects control of their data [45, 46]. This exemption is for research considered of “substantial public interest,” in which case consent is not required. If the research is not considered in the public interest, there are more demanding requirements entailing true anonymization of the data or consent [47].

Private companies now hold enormous quantities of confidential data about their customers, which are sometimes available to academic researchers under DUAs. Kanous and Brock [48] found that agreements used by private data providers were often poorly designed. These agreements were usually derived from non-disclosure or confidentiality agreements that were designed to protect the business secrets of the data provider. Consequently, they are often vague about the nature of the data and the uses permitted to the researcher. Kanous and Brock [48] also found that some agreements include conditions asserting the data provider's right to “derivative” works, which might be interpreted to include analyses and publications. The Yale Open Data Access (YODA) Project was developed to provide independent scientific review of requests to use data created in the private sector [49].

The Accessibility Metadata for Datasets Working Group decided not to attempt to characterize the conditions included in a DUA. Creating an ontology of use conditions was deemed beyond the resources of the group. Fortunately, since the Working Group finished its report, a number of efforts have moved in the direction of ontologies describing the conditions in DUAs, which are detailed below. To accommodate this type of information, the most recent version of DATS was extended with “DataUseCondition” and “ConsentInformation” schemas for referencing dedicated ontologies in anticipation of their future implementation.

## Conditions in Data Use Agreements

Data providers use agreements to ensure that data users behave in ways that protect confidential information and respect consent agreements with research subjects. For example, a common requirement is that researchers will not attempt to re-identify subjects (“safe people”). DUAs used by ICPSR include lists of statistics that should not be published, such as a cell in a cross-tabulation table describing only 1 person, because of re-identification risks. Some agreements require data recipients to submit papers and presentations for review before publication. Standardization of data use conditions would make it easier to automate management and compliance with DUAs, but the diversity and specificity of legacy agreements makes classification difficult. Fortunately, several projects are working on this problem.

Automatable Discovery and Access Matrix (ADA-M) is an ambitious project of the Global Alliance for Genomics and Health (GA4GH) and the International Rare Diseases Research Consortium (IRDiRC) to standardize metadata about data access [50, 51]. ADA-M divides conditions into “permissions” and “terms,” which are arranged under specified concepts. Additional information about permissions and terms can be provided with child fields, and free-text fields are used to capture details and for human readability.

The Data Use Ontology (DUO), which is also a project of GA4GH, is formalizing a controlled vocabulary used by dbGAP for conditions in DUAs for genomic data [52]. DUO is based on the NIH Standard Data Use Limitation (DUL) codes [53]. Data in dbGAP studies are arranged into “consent groups” that share consent agreements and other use conditions. DULs are used to summarize these conditions, although consent groups are often subject to additional conditions not described by DULs. DUL codes are composites combining several types of conditions. For example, General Research Use (GRU), the broadest DUL code, allows studies of statistical methods and population structure or ancestral origin, but the Health/Medical/Biomedical (HMB) code excludes those studies. Like the DUL codes, DUO allows a primary category (e.g., GRU) to be modified by a secondary category (e.g., NMDS [no general methods research]). DUO has been adopted by the European Genome-Phenome Archive [54].

The Informed Consent Ontology (ICO) is being developed to represent terms and relations in informed consent agreements [55, 56]. As the process of obtaining informed consent moves from paper to online systems, it becomes possible to offer individual subjects a wider range of choices about the future use of their data.

The Open Digital Rights Language (ODRL), a recommendation by the World Wide Web Consortium (W3C) [57], provides a rich language to express statements about the use of content and services. It allows the provision of a standard description model and format representing permission, prohibition, and obligation statements. ODRL is recommended in the Data Catalog Vocabulary [17]. Other relevant vocabularies, which also follow a granular approach, are the Open Data Rights Statement Vocabulary [58] and the Agreements ontology (AGR-O) [59].

These vocabularies provide more granularity than DUO and ICO. However, representing DUAs or DULs in a granular way distinguishing permissions, prohibitions, and obligations is a very challenging task, which becomes intractable in many cases because the original documents have not considered such detailed representation and there is ambiguity on choosing relevant terms.

Thus, for cases in which it is not feasible to distinguish between permissions, prohibitions, and obligations, DATS recommends the use of DUO and ICO. However, if an expression relying on ODRL, Open Data Rights Statement (ODRS), or the Agreements ontology can be used, DATS supports pointing to such expression.

**Figure 2:**
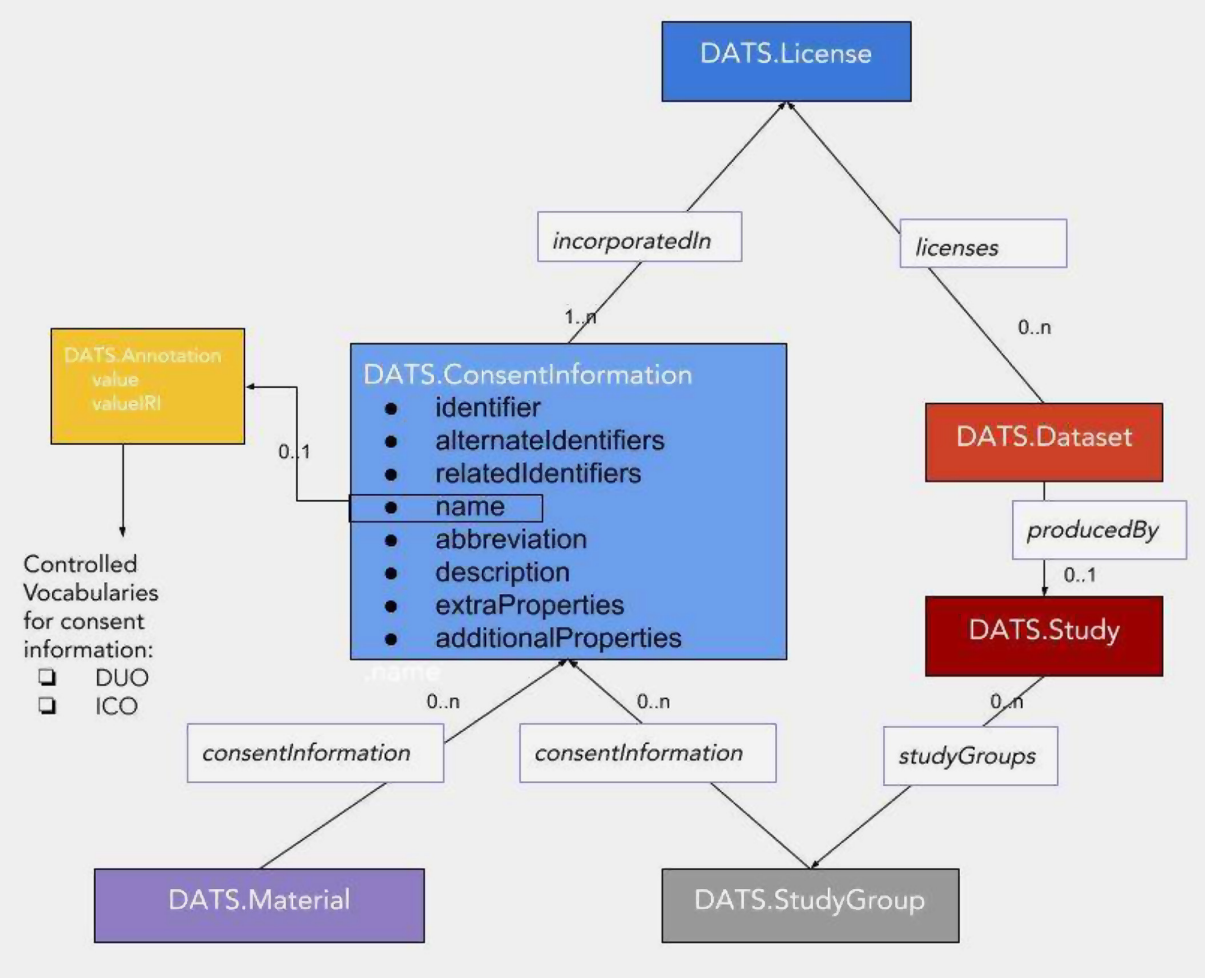
Graphical representation of relevant constructs allowing consent, license, and terms of use information to be made available as information payload in DATS messages. The new "ConsentInformation" schema allows for annotation (semantic markup) with resources such as the Data Use Ontology (DUO; produced by the Global Alliance for Genomic Health) or the Information Consent Ontology (ICO).

The DATS ConsentInformation (consent_info_schema.json) [60] schema has been designed in a flexible way to capture conditions limiting the use of a dataset that may not be included in the DUA. First, as we noted above, the DUA implicitly inherits conditions from all of the previous agreements and approvals covering the data. In particular, the informed consent agreement may include requirements not listed explicitly in the DUA. For this reason, the ConsentInformation schema includes an “incorporatedIn” property that points to the license that it modifies (see Fig. 2).

Second, a research study may include data from subjects who signed different consent agreements. An important example of this regularly occurs in studies that collect genomic data from patients with a specific disease. Some subjects provide consent only to research about their disease, while other subjects allow their data to be used for any type of research. Restrictions of this kind are important to researchers who are searching for data as well as collecting data for reuse (e.g., when creating synthetic cohorts). Dynamic consent creates an even more challenging situation because any subject may give or withdraw consent for a project requesting reuse of their data. To record aspects of dynamic consent, the ConsentInformation schema also has a property for “temporalCoverage,” allowing periods of time when the consent is valid. DATS may be used to describe a specimen or data derived from a tissue sample of a specific individual. To cover these cases the “consentInformation” property may be included in the DATS “material” entity.

Table 4 shows the information in dbGAP for consent groups in the Massachusetts General Hospital (MGH) Atrial Fibrillation Study. The first group (Health/Medical/Biomedical) gave their consent for any type of health, medical, or biomedical research with the exception of studies about the origins or ancestry of individuals or groups. The second group (Disease-Specific) only consented to future research on atrial fibrillation, the focus of the original study. Both consent groups require IRB approval from the recipient's institution. In these cases, DATS can describe multiple “study groups” with different consent conditions and other attributes. In DATS, we would represent each consent group as a StudyGroup with different consentInformation.

**Table 4:** Examples of dbGAP Consent Groups

Consent group	Consent information
Health/Medical/Biomedical (IRB)	Use of this data is limited to health/medical/biomedical purposes, does not include the study of population origins or ancestry. Requestor must provide documentation of local IRB approval. Use of the MGH AF Study data deposited in dbGaP is restricted to research on associations between phenotypes and genotypes. MGH AF Study data may not be used to investigate individual subject genotypes, individual pedigree structures, perceptions of racial/ethnic identity, non-maternity/paternity, and of variables that could be considered as stigmatizing an individual or group. All research must be related to the etiology and prevention of morbidity and mortality of the U.S. population consistent with the demographic distribution in the MGH AF Study. Data users will be required to obtain IRB approval for their projects from their respective institutions (please note that only full or expedited approvals will be accepted).
Disease-Specific (Atrial Fibrillation, IRB, RD)	Use of the data must be related to Atrial Fibrillation and related disorders. Requestor must provide documentation of local IRB approval. Data use is limited to research related to atrial fibrillation and cardiovascular disease.

Source: [61]. MGH AF: Massachusetts General Hospital Atrial Fibrillation.

Third, we expect greater standardization and automation of informed consent agreements to lead to the use of ontologies describing the conditions within these agreements. DATS has a standard way of referring to external ontologies, which can be used for the standards being developed by such projects as ADA-M, DUO, and ICO. By including these conditions in DATS, we allow them to be used for discovery and for filtering search results. Standardization will make it easier to provide this important information to researchers.

## Discussion

There is an inherent tension between the increasing importance of research that combines data from multiple sources and the increasing demand for data that cannot be de-identified. Researchers cannot plan their work unless they know how access will be provided and how long it will take to obtain the necessary permissions. The access metadata objects in the DATS metadata standard differ from other approaches in their focus on the experience of researchers who need to find and intend to reuse existing data. DATS access metadata does not have the level of detail found in metadata standards designed for managing data resources, such as ADA-M [51], Fast Healthcare Interoperability Resources (FHIR) [62], or eXtensible Access Control Markup Language (XACML) [63], but references to other metadata standards can be embedded in DATS. As these and other standards and ontologies develop, data discovery applications will be able to benefit from them through DATS.

Based on the experience garnered through work on DataMed, we are convinced that dividing the access process into 3 steps (authorization, authentication, and type of access) is a useful and original contribution of DATS. New ways of implementing each of these steps are still emerging. Most discussions of data access distinguish between “open” and “restricted” data, but restricted data are distributed in an increasing number of different ways. From a researcher's point of view data that can be downloaded are very different from data that are only accessible on a remote virtual machine. As the bioCADDIE Project has drawn to a close, we document our experience and encourage other organizations to take responsibility for supporting and updating the controlled vocabularies identified by the Accessibility Metadata for Datasets Working Group.

Capturing metadata about the conditions affecting data use will be a time-consuming process until standard ways of describing informed consent and DUAs become part of automated systems for creating and managing research data. There is little standardization in these agreements today, and extracting and classifying the conditions included in legacy agreements is a very complex task. When agreements have been described in standards like ICO, DUO, and ADA-M, they will be searchable and discoverable in DATS. We expect the benefits of automating these agreements to be great but to take time to be realized. Because the technology for electronic health records is developing very quickly, the automation of consent for research use of patient records and tissue samples in FHIR or other standards may be close.

The most difficult problem is obtaining the cooperation of data providers in describing their access and licensing procedures. This does not mean that all data providers must expose metadata about their holdings in DATS. The bioCADDIE Project has demonstrated the flexibility of DATS by mapping and ingesting metadata from >70 data repositories into DataMed [12]. However, the capabilities of data repositories vary widely. Major data repositories (e.g., dbGAP, Protein Data Bank, ICPSR) have established metadata standards, as well as the material and human resources to adapt to new requirements. Other data repositories operate with minimal staff and under precarious funding, even if they serve important scientific communities. We see a great need for NIH and other funding agencies to adopt standards for data repositories, such as the CoreTrustSeal [64], and develop new funding mechanisms designed to provide sustainable support for data curation, dissemination, and preservation. As funding agencies put increased emphasis on FAIR (Findability, Accessibility, Interoperability, Reusability) principles [65], access and data use conditions should become findable as well.

## Availability of supporting materials

The DATS schema is publicly available at https://github.com/datatagsuite.

## Abbreviations

ADA-M: Automatable Discovery and Access Matrix; API: Applications Programming Interface; BD2K: Big Data to Knowledge Program; DATS: Data Tags Suite; dbGaP: Database of Genotypes and Phenotypes; DUA: Data Use Agreement; DUL: Data Use Limitation; DUO: Data Use Ontology; FHIR: Fast Healthcare Interoperability Resources; GA4GH: Global Alliance for Genomics and Health; GDPR: General Data Protection Regulation; GRU: General Research Use; HIPAA: Health Insurance Portability and Accountability Act; ICO: Informed Consent Ontology; ICPSR: Inter-university Consortium for Political and Social Research; IP: Internet Protocol; IRB: institutional review board; NIH: National Institutes of Health; NSF: National Science Foundation; ODRL: Open Digital Rights Language; VINCI: Veterans Affairs Informatics and Computing Infrastructure; W3C: World Wide Web Consortium; XACML: eXtensible Access Control Markup Language.

## Competing Interests

The authors declare that they have no competing interests.

## Funding

This work was supported by the bioCADDIE Project under NIH grant 1U24AI117966-01 and by NIH award OTOD025462. L.O.-M. is funded by NSF OIA-1937136, R01GM118609, and R01HL16835.

## Author contributions

Writing original draft: G.A.; Review and editing: A.G.-B., L.O.-M., P.R.-S.

## Supplementary Material

giz165_GIGA-D-19-00096_Original_Submission

giz165_GIGA-D-19-00096_Revision_1

giz165_Response_to_Reviewer_Comments_Original_Submission

giz165_Reviewer_1_Report_Original_Submission Alex Ball -- 4/9/2019 Reviewed

giz165_Reviewer_2_Report_Original_Submission Thayyaba Khatoon -- 7/20/2019 Reviewed

giz165_Reviewer_3_Report_Original_Submission Greg JanÃ©e -- 9/17/2019 Reviewed
