# Supplementary material for: The Data Tags Suite (DATS) model for discovering data access and use requirements
Source: Gigascience. 2020 Feb 7;9(2):giz165. doi: 10.1093/gigascience/giz165 (PMC7006671; doi:10.1093/gigascience/giz165)
Supplement: giz165_Response_to_Reviewer_Comments_Original_Submission [file giz165_response_to_reviewer_comments_original_submission.pdf]

Author's Response To Reviewer Comments

Close

Our responses to reviewers are in a file submitted with the revised manuscript.

Close
