# Supplementary material for: The Data Tags Suite (DATS) model for discovering data access and use requirements
Source: Gigascience. 2020 Feb 7;9(2):giz165. doi: 10.1093/gigascience/giz165 (PMC7006671; doi:10.1093/gigascience/giz165)
Supplement: giz165_Reviewer_1_Report_Original_Submission — Alex Ball -- 4/9/2019 Reviewed [file giz165_reviewer_1_report_original_submission.pdf]

Reviewer Report

Title: The Data Tags Suite (DATS) Model for Discovering Data Access and Use

Version: Original Submission **Date:** 4/9/2019

Reviewer name: Alex Ball

Reviewer Comments to Author:

This technical note provides a detailed look at the issues of access and usage conditions associated with datasets and the approach taken with DATS for handling them.

I think the paper would benefit from a little more context, and a little more clarity over the technical implementation. The third paragraph of the introduction puts a lot of weight on its first two references to explain DATS, but it would help to import some details into the current paper, such as a potted version history of DATS, and when the elements discussed in this paper were added and by whom (e.g. access terms, in v2.0, by WG7); this would help make sense of certain aspects being described as "new", implying other parts are not as new, but presumably still new enough to warrant discussion in this paper. Comments like "the most recent version of DATS was extended with 'dataUseCondition' and 'consentInformation' schemas" (p.11, l.28) lacks a degree of precision, requiring the reader to dig around to find where the version information is (https://github.com/biocaddie/WG3-MetadataSpecifications/releases, not referenced) and compare against the submission date...

Similarly in figure 2 we get a useful diagram of how ConsentInformation fits into the general schema, but there is nothing to say where the access information goes (nor the data use conditions) and in what form the information is recorded. We have tables of types/methods, of course, but are these controlled terms in a vocabulary, or elements that decompose further, or what?

In the first paragraph of the section "DATS Use Metadata" there are a few things I would question. "Creative Commons, which is best known for its copyright licenses, also provides licenses for data." Well no, it does not *also* provide licences for data; its regular licences since version 4.0 have *also been suitable* for data, and regarding the content of the reference, CC0 is not purely a data licence/waiver, but can be used for anything. The rest of the paragraph strongly implies that "licence" is synonymous with "data use agreement" and while I am not a lawyer, I do not believe this to be the case. It is in the nature of licences to grant permissions to the licensee to do things not permitted by default under the law, whereas agreements may also impose greater restrictions than the default (for example by forbidding things that might be permitted under fair use or fair dealing exceptions). There is also an implication that intellectual property law is irrelevant to data, which it is not.

The issue of dynamic consent is an interesting one from an implementation perspective. Presumably this, like the technical aspects of authentication, is out of scope (which is a shame), but perhaps you could explicitly link it to your discussion of per-participant consent (p.13, l.45f) to underline the implications it has at the point of access.

Regarding data use conditions, the development of clear ontologies certainly opens up a lot of promising possibilities. I wonder if you have any reflections on whether these ontologies can fully capture the nuance of legal wording in data use agreements, and if not, whether conditions written using these

ontologies should be considered approximate (non-normative) descriptions of the conditions or alternative legally binding (normative) conditions in their own right. Towards the end you suggest using the ontologies directly within the written agreements: do you think they are ready to carry that kind of legal weight (i.e. they are expressed in legally enforceable wording)?
More trivial copy editing remarks:
- p.4, l.13: In "to be a focus in work of", are you missing a "the" after "in"?
- p.4, l.56: There is a slightly jarring use of first person in "We also have a range". You could keep things in third person by saying, for example, "Providers can select from a range" or "Providers have at their disposal a range"
- At p.4, l.57 and p.5, l.32 you talk about imposing costs on researchers. This could easily be taken literally or figuratively; if meant figuratively, it might be clearer to talk in terms of effort or (using a different metaphor) burdens, barriers or investment.
- p.6, ll.5, 38, 23: You cross-reference a discussion of licences, but even though DATS has a schema for licences, the discussion I presume you mean is headed "DATS Use Metadata", and in it you say you prefer "data use agreement" to "license" and thereafter avoid the L word.
- p.9, l.17: "all future research": not literally all future research, but all further research based on these data.

Level of Interest

Please indicate how interesting you found the manuscript: Choose an item.

Quality of Written English

Please indicate the quality of language in the manuscript: Choose an item.

Declaration of Competing Interests

Please complete a declaration of competing interests, considering the following questions:

- Have you in the past five years received reimbursements, fees, funding, or salary from an organisation that may in any way gain or lose financially from the publication of this manuscript, either now or in the future?
- Do you hold any stocks or shares in an organisation that may in any way gain or lose financially from the publication of this manuscript, either now or in the future?
- Do you hold or are you currently applying for any patents relating to the content of the manuscript?
- Have you received reimbursements, fees, funding, or salary from an organization that holds or has applied for patents relating to the content of the manuscript?
- Do you have any other financial competing interests?
- Do you have any non-financial competing interests in relation to this paper?

If you can answer no to all of the above, write 'I declare that I have no competing interests' below. If your reply is yes to any, please give details below.

I declare that I have no competing interests

I agree to the open peer review policy of the journal. I understand that my name will be included on my report to the authors and, if the manuscript is accepted for publication, my named report including any attachments I upload will be posted on the website along with the authors' responses. I agree for my report to be made available under an Open Access Creative Commons CC-BY license (http://creativecommons.org/licenses/by/4.0/). I understand that any comments which I do not wish to be included in my named report can be included as confidential comments to the editors, which will not be published.

Choose an item.

To further support our reviewers, we have joined with Publons, where you can gain additional credit to further highlight your hard work (see: https://publons.com/journal/530/gigascience). On publication of this paper, your review will be automatically added to Publons, you can then choose whether or not to claim your Publons credit. I understand this statement.

Yes Choose an item.
