# Supplementary material for: The Data Tags Suite (DATS) model for discovering data access and use requirements
Source: Gigascience. 2020 Feb 7;9(2):giz165. doi: 10.1093/gigascience/giz165 (PMC7006671; doi:10.1093/gigascience/giz165)
Supplement: giz165_Reviewer_3_Report_Original_Submission — Greg JanÃ©e -- 9/17/2019 Reviewed [file giz165_reviewer_3_report_original_submission.pdf]

Reviewer Report

Title: The Data Tags Suite (DATS) Model for Discovering Data Access and Use

Version: Original Submission **Date:** 9/17/2019

Reviewer name: Greg JanÃ©e

Reviewer Comments to Author:

This paper comes from the bioCADDIE working group that authored the accessibility portion of DATS, and the paper describes some of the background research and rationale that went into the selection and development of the accessibility-related DATS elements. Because DATS has already been published, the new material here is simply the description of the development process, not the DATS elements themselves. This information is useful because it is not to be found anywhere in the DATS documentation. The paper helpfully expands on the meanings of accessibility-related controlled vocabularies, though, arguably, term definitions really belong in the DATS documentation.
The title of the paper could be improved. "Discovering ... using DATS" leads the reader to believe they will learn how to use DATS to discover something. But as noted, this paper is describing the development of DATS. I would suggest a title along the lines of "Development of the DATS access and use requirements".
In Figure 1, the meaning of the red dashed lines is not defined. It would also be helpful if the relationships between the entities were annotated.
In Figure 2, the red underlining of certain words appears to have no significance, and instead appears to be left over from a spell checker.
Grammatical error, first paragraph, second sentence: "This is only possible if data can be discovered, accessed and MADE amenable to reuse."

Level of Interest

Please indicate how interesting you found the manuscript: Choose an item.

Quality of Written English

Please indicate the quality of language in the manuscript: Choose an item.

Declaration of Competing Interests

Please complete a declaration of competing interests, considering the following questions:

- Have you in the past five years received reimbursements, fees, funding, or salary from an organisation that may in any way gain or lose financially from the publication of this manuscript, either now or in the future?

- Do you hold any stocks or shares in an organisation that may in any way gain or lose financially from the publication of this manuscript, either now or in the future?
- Do you hold or are you currently applying for any patents relating to the content of the manuscript?
- Have you received reimbursements, fees, funding, or salary from an organization that holds or has applied for patents relating to the content of the manuscript?
- Do you have any other financial competing interests?
- Do you have any non-financial competing interests in relation to this paper?

If you can answer no to all of the above, write 'I declare that I have no competing interests' below. If your reply is yes to any, please give details below.

I declare that I have no competing interests

I agree to the open peer review policy of the journal. I understand that my name will be included on my report to the authors and, if the manuscript is accepted for publication, my named report including any attachments I upload will be posted on the website along with the authors' responses. I agree for my report to be made available under an Open Access Creative Commons CC-BY license (http://creativecommons.org/licenses/by/4.0/). I understand that any comments which I do not wish to be included in my named report can be included as confidential comments to the editors, which will not be published.

Choose an item.

To further support our reviewers, we have joined with Publons, where you can gain additional credit to further highlight your hard work (see: https://publons.com/journal/530/gigascience). On publication of this paper, your review will be automatically added to Publons, you can then choose whether or not to claim your Publons credit. I understand this statement.

Yes Choose an item.
